# Electromyographic Analysis of Back Muscle Activation During Lat Pulldown Exercise: Effects of Grip Variations and Forearm Orientation

**DOI:** 10.3390/jfmk10030345

**Published:** 2025-09-11

**Authors:** Andrea Buonsenso, Domenico Di Fonza, Gloria Di Claudio, Massimiliano Carangelo, Marco Centorbi, Alessandra di Cagno, Giuseppe Calcagno, Giovanni Fiorilli

**Affiliations:** 1 Department of Theoretical and Applied Sciences, eCampus University, 22060 Novedrate, Italy; andrea.buonsenso@uniecampus.it; 2 Department of Medicine and Health Sciences, University of Molise, 86100 Campobasso, Italy; difonzad@gmail.com (D.D.F.); gloria.diclaudio@univr.it (G.D.C.); massimilianocarangelo@gmail.com (M.C.); giuseppe.calcagno@unimol.it (G.C.); fiorilli@unimol.it (G.F.); 3 Department of Neurosciences, Biomedicine and Movement, University of Verona, 37129 Verona, Italy; 4 Department of Movement, Human and Health Sciences, University of Rome “Foro Italico”, 00135 Rome, Italy; alessandra.dicagno@uniroma4.it; 5 Department of Human Sciences, Guglielmo Marconi University, 00193 Rome, Italy

**Keywords:** EMG, back muscle, muscle activity, resistance training

## Abstract

**Objectives**: The lat pulldown machine is one of the most versatile pieces of equipment for back strengthening, allowing variations in grip and load. However, there are significant gaps in the literature regarding the relationship between exercise modality and specific muscle activation. **Methods**: This study examined the electromyographic (EMG) activity of major back muscles during seven lat pulldown exercise variants that differed in grip type, width, and trunk inclination. Forty male subjects, with at least 5 years of resistance training experience, performed five repetitions of lat pulldown exercise using 70% of their repetition maximum. Prior to the surface EMG analysis, maximal voluntary contraction (MVC) tests were performed for each muscle group analysed, specifically the latissimus dorsi, posterior deltoid, brachial biceps, middle and lower trapezium, and infraspinatus. The normalised root mean square of the EMG (NrmsEMG) activity for each muscle was recorded during full, concentric, and eccentric movements. **Results**: Multivariate analysis of variance (MANOVA) showed no significant difference in the NrmsEMG muscle activation across the different lat pulldown exercise variations (all *p* > 0.05). A significant difference was found in the posterior deltoid where the wide-pronated grip with a 30° trunk inclination showed greater EMG activation compared to the wide pronated grip (*p* = 0.011) and wide neutral grip (*p* = 0.017). **Conclusions**: These findings suggest that grip variations may not significantly alter latissimus dorsi recruitment, challenging the assumption that grip effectiveness targets this muscle. The results highlight the need for individualised approaches to exercise selection, given the variability in muscle activation patterns observed.

## 1. Introduction

Back muscle training plays a fundamental role in both athletic performance and rehabilitation, contributing to enhanced strength and the prevention of musculoskeletal injuries [1]. Being among the most widely utilised exercises for strengthening this anatomical region, the lat pulldown exercise stands out for its versatility, offering the ability to modulate load parameters and vary grip configurations to selectively target specific muscle groups [2]. The primary muscle targeted during lat pulldown is the latissimus dorsi (LD), a large, fan-shaped muscle responsible for shoulder extension, adduction, and internal rotation. However, depending on the execution technique and body position, this exercise also activates several synergistic and stabilising muscles [3], including the middle (MT) and lower trapezius (LT), posterior deltoid (PD), infraspinatus (IN), and biceps brachii (BB) [4,5]. Understanding how these muscles are selectively recruited under different exercise configurations is crucial for tailoring training protocols to specific performance and therapeutic objectives. One of the key variables influencing muscle recruitment during resistance exercise is grip configuration, which encompasses grip type (pronated, supinated, or neutral), grip width (narrow vs. wide), and forearm orientation relative to trunk position [6]. Variations in these parameters can alter shoulder and scapular biomechanics, thereby modifying the mechanical loading experienced by different muscle groups [7]. Despite the widespread use of these variations in resistance training, the scientific literature remains inconclusive and fragmented regarding their specific impacts on back muscle activation. Surface electromyography (sEMG) is one of the most extensively employed tools for analysing muscle activation during resistance exercises, providing quantitative data on muscle activity intensity and differential activation patterns among various muscle groups [8]. Several studies have employed sEMG to assess muscle activation patterns across various lat pulldown exercise variations, although each presents certain limitations or lacks evaluation of key biomechanical factors. Sperandei et al. (2009) [8] investigated muscle activity during lat pulldown exercises performed behind the neck, in front of the neck, and using a V-bar attachment. However, this study did not account for the potential influence of grip type and forearm orientation, which may critically affect the neuromuscular recruitment. Lusk et al. (2010) [9] evaluated LD, MT, and BB activation during wide-pronated, wide-supinated, narrow-pronated, and narrow-supinated grips. Similarly, Signorile et al. (2002) [10] investigated the effects of different hand positions (closed grip, supinated grip, wide grip anterior, and wide grip posterior) on the posterior deltoid, latissimus dorsi, pectoralis major, teres major, and long head of the triceps during the lat pulldown. Lehman et al. (2004) [11] evaluated the LD, BB and MT/rhomboid muscle groups activation during the wide grip pulldown, reverse grip pulldown, and seated row with and without non-retracted scapula. All these studies considered neutral grip positions, trunk inclination, and comprehensive electrode placement on the key synergists.

Given the identified gaps in the existing literature, the present study aimed to evaluate electromyographic activity of major back muscles—including the LD, MT, LT, PD, IN, and BB—across seven different lat pulldown exercise variants. These variants differ in terms of grip type (pronated, supinated, or neutral), grip width (narrow or wide), and trunk inclination (upright or 30° inclined angle).

## 2. Materials and Methods

### 2.1. Study Design

This observational study was designed to evaluate electromyographic activation of the major dorsal muscle groups during lat pulldown exercise to assess whether there is a plug that allows greater activation of the LD. The effects of three biomechanical variables were analysed: grip type (pronated, supinated, or neutral), grip width (narrow or wide), and trunk inclination (vertical or 30° inclination).

#### 2.1.1. Participants

Forty physically active male participants (age: 23.88 ± 3.56 years) were recruited for the present study. The participants had trained for at least five years, with a training frequency of at least three times per week. The anthropometric characteristics of the participants are shown in Table 1.

The inclusion criteria were as follows: (a) age between 18 and 35 years, (b) minimum five years of resistance training experience, (c) specific familiarity with the lat pulldown exercise, and (d) absence of upper limb musculoskeletal injuries in the previous six months. All participants were thoroughly informed about the experimental procedures and provided written informed consent prior to the assessments. The research protocol was approved by the Local Bioethical Committee of the University of Molise (6306/2025) and was conducted in accordance with the principles of the Declaration of Helsinki for research involving human participants.

#### 2.1.2. Experimental Procedure

**Figure 1:**
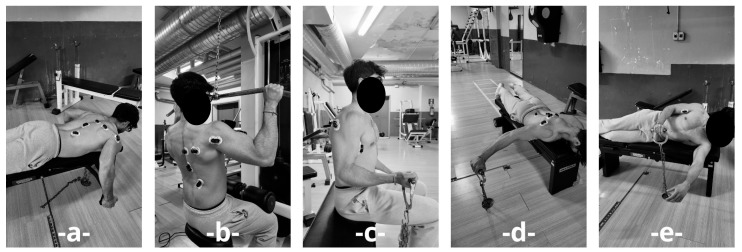
Isometric tests to calculate the maximal voluntary contractions (MVCs) for each muscle group. (**a**) row with a neutral grip “trazy” bar in a prone position; (**b**) 90° lat pulldown; (**c**) 90° biceps curl in a seated position; (**d**) 90° reverse fly; (**e**) shoulder external rotation in a side-lying position.

Participants were asked to avoid strenuous workouts, especially for the back, in the three days before the evaluation tests and experimentation. The participants underwent a standardised anthropometric assessment to measure their height and body mass. Subsequently, the participants were asked to perform maximal voluntary contraction (MVCs) for each muscle group. The MVC value was defined as the peak EMG activation recorded during the five trials for each muscle. All MVCs were performed as isometric contractions for 5 s. For each muscle, five maximal contractions were executed, with 60 s recovery intervals between each effort. The order of execution was randomised to minimise fatigue effects [11,12]. The exercises were performed as follows: (1) in a prone position on a bench, participants performed a row using a neutral grip “trazy” bar; (2) a lat pulldown was executed with a biacromial grip, maintaining a 90° angle between the humerus and forearm; (3) in a seated position, participants performed a unilateral biceps curl, keeping the humerus and forearm at a 90° angle; (4) while prone, participants performed a reverse fly, maintaining a 90° angle between the humerus and torso; (5) finally, in a side-lying position on a flat bench, participants executed a shoulder external rotation. Peak activation of each muscle group analysed was considered regardless of the specific test performed. The MVCs test is shown in Figure 1.

In the subsequent session, following a standardised warm-up, the participants performed seven lat pulldown variants in a randomised order: (a) Wide pronated grip lat pulldown (WPG) (1.5× biacromial distance) (b) Narrow pronated grip lat pulldown (NPG) (biacromial distance) (c) Supinated grip lat pulldown (SG) (biacromial distance) (d) Wide pronated grip lat pulldown with 30° trunk inclination (WPG30°) (e) Narrow neutral grip lat pulldown (NNG) (biacromial distance) (f) Wide neutral grip lat pulldown with 30° trunk inclination (WNG30°) (g) Wide neutral grip lat pulldown (WNG) (1.5× biacromial distance). The biacromial distance was measured as the distance between the right and left acromion processes using an anthropometric calliper. For each variant, the participants performed five consecutive repetitions using a load corresponding to 70% of their 1RM, which was determined during a familiarisation session.

**Figure 2:**
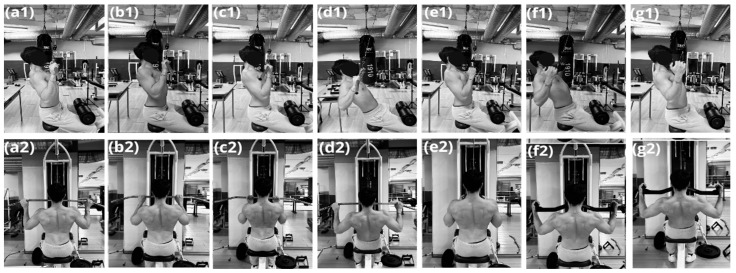
Lat pulldown variants execution. (**a**) Wide pronated grip lat pulldown (WPG) (1.5× biacromial distance) (**b**) Narrow pronated grip lat pulldown (NPG) (biacromial distance) (**c**) Supinated grip lat pulldown (SG) (biacromial distance) (**d**) Wide pronated grip lat pulldown with 30° trunk inclination (WPG30°) (**e**) Narrow neutral grip lat pulldown (NNG) (biacromial distance) (**f**) Wide neutral grip lat pulldown with 30° trunk inclination (WNG30°) (**g**) Wide neutral grip lat pulldown (WNG) (1.5× biacromial distance).

Each participant received specific technical instructions from a certified trainer. The trainer carefully observed each repetition to verify compliance with the study guidelines. Particular attention was paid to the execution speed, which was tracked using the time indicators provided by real-time data acquisition software (Model XB700; Franz Mfg. Co. Ink, East Haven, CT, USA). The execution tempo was standardised at 2 s for the concentric phase (pull) and 2 s for the eccentric phase (controlled release), following an auditory beep and a visual flash of a metronome. A 3 min recovery interval was observed between the different experimental conditions to prevent muscle fatigue. The execution sequence of the variants was random. The grip and execution movements are shown in Figure 2.

### 2.2. EMG Measurement and Analysis

Electromyographic activity was recorded using surface electromyography (sEMG) with a Cometa wireless system (Wavex Wireless EMG System; Cometa srl, Milan, Italy; sampling frequency of 2000 Hz). Electrodes were positioned on the following muscle groups: LD, MT, LT, PD, BB, and IN. Electrode placement was performed according to the recommendations of the Surface ElectroMyoGraphy (SENIAM) project [13]. To enhance the EMG signal quality, the skin at each electrode site was gently abraded with fine sandpaper and cleaned with an alcohol wipe. Self-adhesive surface electrodes with a diameter of 1 cm were applied to the target muscle groups. The EMG signal was acquired at a sampling frequency of 2000 Hz and subsequently filtered using a low-pass filter with a cut-off frequency of 450 Hz and a high-pass filter with a cutoff frequency of 10 Hz, followed by signal rectification and smoothing using a symmetrical window of 100 Hz. Consistent with earlier studies [9,11], the root mean square (rmsEMG) of each EMG signal was used to assess the mean level of muscle activation during the full range of motion for each repetition, taking the average of five repetitions for each grip and trunk inclination angle. For each participant and experimental condition, the rmsEMG values were normalised relative to those obtained during the MVC tests (NrmsEMG). The level of NrmsEMG for each muscle reflects the percentage of maximal activation and indicates the relative involvement of each muscle during each analysed movement. NrmsEMG was used for the analysis.

### 2.3. Statistical Analysis

Statistical analysis was performed using SPSS Statistics 21 software (IBM, Armonk, NY, USA) to analyse the differences in muscle activation during the different lat machine grips. The normal distribution of continuous variables was verified using the Shapiro–Wilk test. Descriptive statistics are presented as mean and standard deviation. Multivariate analysis of variance (MANOVA) was used to test the differences in NrmsEMG muscle group activation, both across the full movement and when the movement was divided into concentric and eccentric phases, considered as dependent variables, in each grip technique during the lat pulldown exercise, considered an independent variable. Bonferroni post hoc corrections were performed to identify the differences among the grips. The alpha test level for statistical significance was set at *p* < 0.05.

## 3. Results

**Figure 3:**
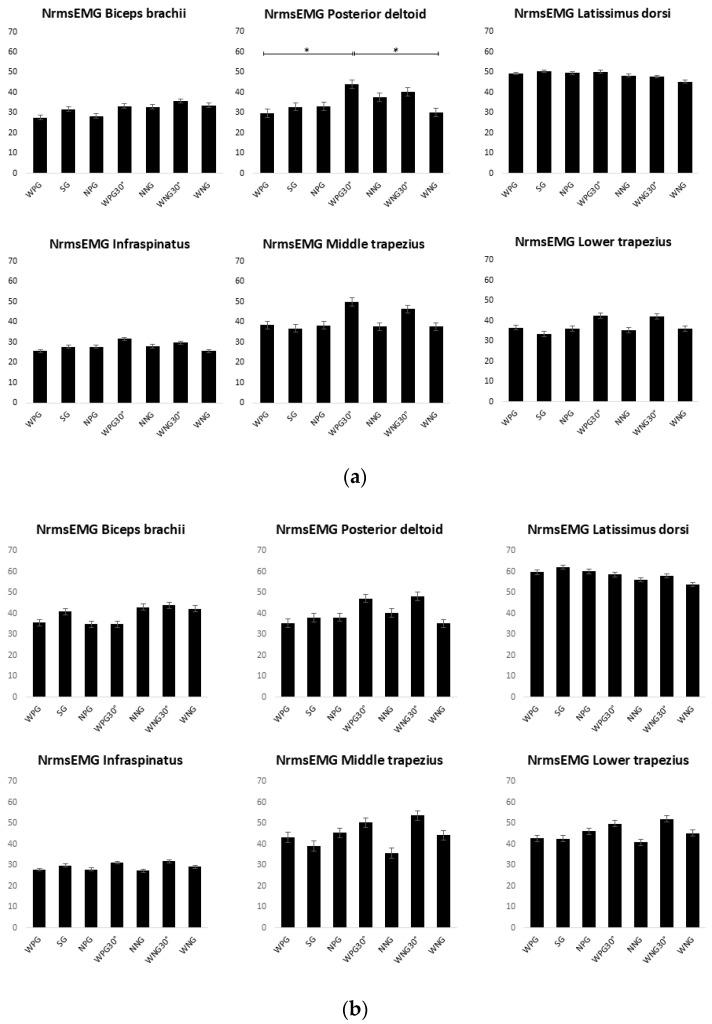

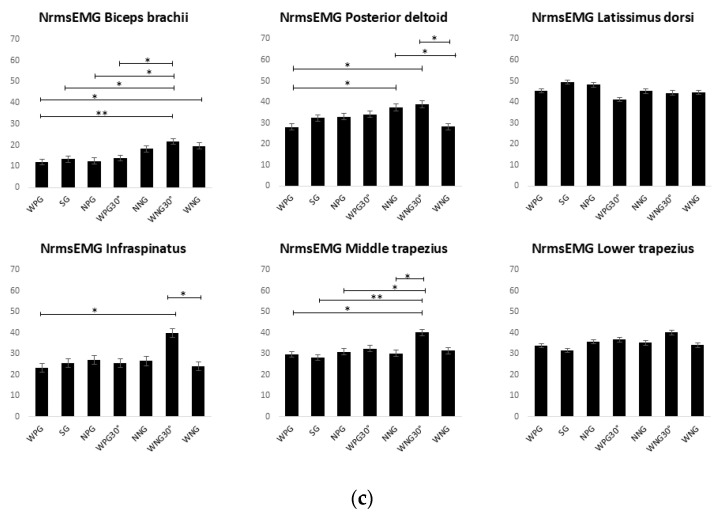
(**a**) NrmsEMG of each muscle group during the seven grips. (**b**) NrmsEMG in the concentric phase. (**c**) NrmsEMG in the eccentric phase. WPG: wide pronated grip; SG: supinated grip; NPG: narrow prone grip; WPG30°: wide prone grip with 30° trunk inclination; NNG: narrow neutral grip; WNG30°: wide neutral grip with 30° trunk inclination; WNG: wide neutral grip; *: *p* < 0.05; ** *p* < 0.01.

MANOVA performed during full movement analysis showed no significant differences in LD (F(1,6) = 0.415, *p* = 0.868), MT (F(1,6) = 0.919, *p* = 0.484), LT (F(1,6) = 1.225, *p* = 0.298), BB (F(1,6) = 1.023, *p* = 0.414), and IN (F(1,6) = 0.500, *p* = 0.807) muscle activation during different grip–lat pulldown exercises. Only the PD (F(1,6) = 3.621, *p* = 0.002) showed higher NrmsEMG activity during the WPG30° than during the WPG (*p* = 0.011) and WNG lat pulldown (*p* = 0.017). The detailed results of the NrmsEMG during each grip and during full, concentric, and eccentric movements are shown in Figure 3a–c, respectively.

**Figure 4:**
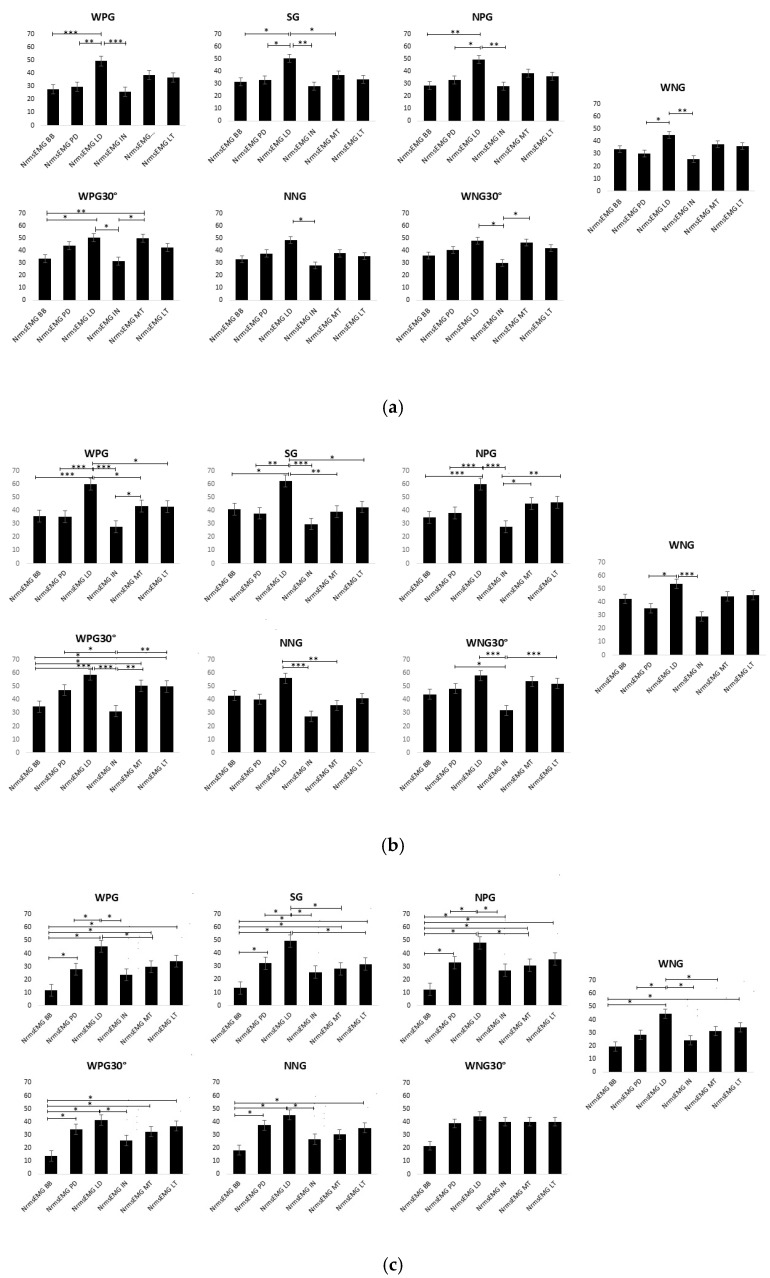
(**a**). NrmsEMG during each grip. (**b**). NrmsEMG in the concentric phase. (**c**). NrmsEMG in the eccentric phase. WPG: wide pronated grip; SG: supinated grip; NPG: narrow prone grip; WPG30°: wide prone grip with 30° trunk inclination; NNG: narrow neutral grip; WNG30°: wide neutral grip with 30° trunk inclination; WNG: wide neutral grip; BB: biceps brachii; PD: posterior deltoid; LD: latissimus dorsi; IN: infraspinatus; MT: middle trapezius; LT: lower trapezius; *: *p* < 0.05; **: *p* < 0.01; ***: *p* < 0.001.

Finally, by comparing the individual muscle activations for each grip, the MANOVA performed during the full movement analysis showed that the LD exhibited greater activation than several other muscle groups across different grip variations. The detailed results of the NrmsEMG during each grip and during full, concentric, and eccentric movements are shown in Figure 4.

## 4. Discussion

A comprehensive analysis of muscle activation during lat pulldown exercise using sEMG addresses a significant gap in the existing literature. By exploring various grip types, forearm orientations, and trunk inclinations, this study provides valuable insights into aspects that have previously been overlooked. To our knowledge, this is the first study to evaluate all grip variations and forearm orientations during the lat pulldown exercise on the main muscle groups involved. The main result of this study was that no significant differences were found in LD activation across the seven exercise variants, including both concentric and eccentric phases, indicating that none of the grip configurations produced superior target muscle activation. This highlights a potential limitation in the generalisability of sEMG data across individuals and underscores the complex interplay between biomechanics and motor control strategies. The lack of statistically significant findings may be primarily attributed to substantial interindividual variability in neuromuscular responses. Despite rigorous control of experimental conditions, including standardised load, movement cadence, and participant training background, variations in anatomical structure, motor recruitment patterns, and neuromuscular efficiency likely contributed to the divergent muscle activation profiles. Three studies [9,10,11] reported variable results regarding grip-specific muscle activation during the lat pulldown exercise. Lusk et al. (2010) [9], by comparing four grip variations during lat pulldown, showed a higher activation of the LD during the pronated grip than that during the SG grip. Signorile et al. (2002) [10] found that grip variations affect the activities of specific muscle, highlighting that wide grip anterior produces greater activation of LD. Lehman et al. (2004) [11] found that the wide grip pulldown and the seated row showed the greatest level of LD activation relative to the BB. However, these studies are not without methodological limitations: small sample sizes (10 and 12 participants), heterogeneity of participants’ ages included in the studies, and relatively short training experience used as an inclusion criterion (6 months to 1 year). These factors may have influenced the outcomes, as novice participants, although instructed, lacked sufficient familiarity with the required motor task. In addition, the authors reported that different variations in LD exercises can lead to only slight alterations in the muscle activation patterns of the main muscles involved. While our findings align with their conclusion that grip type alone does not significantly alter LD activation, they expand upon this by demonstrating that even with added biomechanical complexity, no consistent pattern of increased LD engagement would be observed. This may indicate a ceiling effect of motor recruitment under submaximal loads in well-trained subjects.

In our study, when examining full-movement lat pulldown exercises, muscle activation patterns across different grip variations showed a significant difference between the LD and other muscles, such as the BB, PD, and IN. The LD consistently demonstrated relatively stable activation levels, regardless of grip configuration, from 45% to 50% of the MVC, likely because of its primary role as an agonist in shoulder adduction and extension during vertical pulling movements. Based on our analysis, we conclude that, irrespective of grip type and orientation, the LD consistently remains the primary muscle targeted during the lat pulldown exercise. In contrast, the BB, which functions as a synergist and is highly responsive to elbow positioning and forearm orientation, exhibits greater variability in activation, particularly in supinated grips, where mechanical leverage is enhanced [14]. The PD and IN, which act as stabilisers and contributors to shoulder extension and external rotation, are more sensitive to changes in trunk inclination and scapular positioning, which can shift depending on grip type and width [15]. This differential responsiveness highlights the distinct biomechanical roles of these muscles and suggests that, while the LD maintains consistent activation across grips, the accessory muscles adapt dynamically to postural and kinematic alterations. Supporting this, EMG analyses have shown significantly increased activation in the PD during the WPG30° compared to the WPG and WNG, whereas latissimus dorsi activation remained unchanged [16]. These findings underscore the importance of individualised exercise selection, as modifying grip parameters may preferentially target synergistic musculature without substantially altering LD activation. Although the use of NrmsEMG provides a reliable method for the quantification of the magnitude of muscle activation, this metric alone may overlook relevant aspects of intermuscular coordination. For example, as illustrated in Figure 4, the WPG30° and WNG30° conditions exhibited a more balanced distribution of activation between the latissimus dorsi (LD) and the middle trapezius (MT), whereas in other grip variations, the LD clearly dominated. These relative differences suggest that trunk inclination and grip orientation may influence not only the absolute activation level of each muscle but also their relative contribution to the pulling movement. Therefore, future research should go beyond amplitude-based indices and incorporate analytical approaches, such as co-activation ratios, muscle synergy analysis, or time–frequency domain methods, to better characterise the neuromuscular strategies underlying lat pulldown execution.

When examining the full movement divided into concentric and eccentric phases, the first revealed no significant differences in the activation levels of the muscles analysed across the various grips of the lat pulldown exercise. This finding is consistent with that of Sperandei et al. (2009) [8], who observed that changes in grip configuration did not significantly affect muscle recruitment patterns during the concentric portion, suggesting that grip may have a greater influence on joint kinematics than neuromuscular output. In addition, the analysis confirmed the findings observed during the full movement results, demonstrating higher activation of the LD than the other muscle groups in all grip variations. Moreover, LD activity reached a greater percentage of MVS, ranging from 56% to 62%.

Similarly, the eccentric phase analysis showed no significant differences in the activation levels of LD across the various grips. This confirms the primary role of the LD during both the concentric and eccentric phases of the lat pulldown exercise. In addition, a substantial reduction in BB activity was observed compared to its own activity during the concentric phase, revealing a reduced activation of the BB compared to the primary muscle groups involved, such as the PD, MT, LD, and LT. This lower level of BB activity may be attributed to its diminished role in stabilisation and control during the eccentric portion of the movement. These findings are consistent with prior research showing that BB activation significantly declines during eccentric lat pulldown phases, whereas stabilising muscles, such as the trapezius and posterior deltoid, maintain or increase their contribution [8,9]. Interestingly, the use of neutral grips appeared to attenuate the reduction in BB activation, possibly due to altered forearm orientation, which facilitates elbow flexor recruitment [9]. Additionally, greater activation of the IN and MT muscles was noted in the WNG30° grip condition compared to the other grip configurations, suggesting a compensatory stabilising function in response to the biomechanical demands of the altered shoulder angle. This is supported by several studies that have found increased activation of the IN and TM muscles when using wider and neutral grip variations [15], and an increased posterior shoulder muscle activity during grips that shift load distribution or increase instability [17]. No significant differences were observed in the WNG30° condition, which may be explained by the increased need for muscular stabilisation owing to altered biomechanics. The neutral grip combined with trunk inclination appears to be less favourable for LD activation, increasing the co-activation of scapular stabilisers [18]. Conversely, this result was not evident in the WPG30° condition, where internal rotation of the humerus could compromise scapular stability and control, reducing the activation of stabilising muscles, such as the IN and MT [19].

The research methodology, which includes the use of surface EMG and MVC tests, provides a robust framework for analysing muscle activation patterns. However, the limitations of this study, such as the relatively small sample size and focus on male participants, should be considered when interpreting these results. The lack of consideration of the limb length of each participant may have influenced muscle activation. This study did not consider the differences in activation duration and timing of synergistic muscle groups during different exercise conditions. No analysis of spectral features has been considered; however, muscle activation in the participants frequently remained below 60% of the MVC. This relatively modest activation level suggests that high-threshold motor units, particularly those associated with Type II fast-twitch fibres, may not be engaged at this intensity. Overall, this study contributes valuable insights to the field of resistance training and biomechanics, highlighting the complexity of muscle activation during lat pulldown exercises and the need for personalised training approaches to optimise back muscle development and strength in the future. 

## 5. Conclusions

Varying the grip type or forearm orientation did not significantly change the activation of the main muscles involved in the lat pulldown movement. In addition, LD activation remained relatively stable for each variant. These findings challenge the conventional assumption regarding the effectiveness of different grip types in maximising LD stimulation. 

Pragmatically, as grip type and forearm orientation did not significantly influence latissimus dorsi activation, practitioners and strength coaches may prioritise the grip variation that ensures the best comfort, joint safety, and technical control for each individual. Nevertheless, given the interindividual variability observed in muscle recruitment, coaches should monitor athletes’ subjective feedback and execution quality, tailoring grip and trunk inclination to optimise training effectiveness and reduce the risk of injury.

## Figures and Tables

**Table 1:** Sample characteristics.

**Variable**	**Mean ± SD**
**Number of Participants**	40
**Age**	23.88 ± 3.56
**Body Mass (kg)**	77.49 ± 7.17
**Height (cm)**	176.27 ± 5.36
**BMI**	23.70 ± 1.85

SD: standard deviation; kg: kilograms; cm: centimetre; BMI: body max index.

## Data Availability

Data will be made available on request.

## Abbreviations

MVC	Maximal Voluntary Contraction
sEMG	Surface Electomyography
rmsEMG	Root Mean Square Electromyography
NrmsEMG	Normalised Root Mean Square of the electromyography
WPG	Wide Pronated Grip
NPG	Narrow Prone Grip
SG	Supinated Grip
WPG30°	Wide Pronated Grip with 30° trunk inclination
NNG	Narrow Neutral Grip
WNG30°	Wide Neutral Grip with 30° trunk inclination
WNG	Wide Neutral Grip
LD	Latissimus Dorsi
MT	Middle Trapezius
LT	Lower Trapezius
PD	Posterior Deltoid
BB	Biceps Brachii
IN	Infraspinatus
